# *Echinostoma revolutum* Infection in Children, Pursat Province, Cambodia

**DOI:** 10.3201/eid1701.100920

**Published:** 2011-01

**Authors:** Woon-Mok Sohn, Jong-Yil Chai, Tai-Soon Yong, Keeseon S. Eom, Cheong-Ha Yoon, Muth Sinuon, Duong Socheat, Soon-Hyung Lee

**Affiliations:** Author affiliations: Gyeongsang National University School of Medicine, Jinju, South Korea (W.-M. Sohn);; Seoul National University College of Medicine, Seoul, South Korea (J.-Y. Chai, S.-H. Lee);; Korea Association of Health Promotion, Seoul (J.-Y. Chai, C.-H. Yoon);; Yonsei University College of Medicine, Seoul (T.-S. Yong);; Chungbuk National University College of Medicine, Cheongju, South Korea (K.S. Eom);; Center for National Malaria Control, Phnom Penh, Cambodia (M. Sinuon, D. Socheat)

**Keywords:** Parasites, Echinostoma revolutum, echinostomiasis, trematode, prevalence, children, Cambodia, dispatch

## Abstract

To determine the prevalence of helminthic infections in Pursat Province, Cambodia, we tested fecal specimens from 471 children, 10–14 years of age, in June 2007. The prevalence of infection with echinostome flukes ranged from 7.5% to 22.4% in 4 schools surveyed. Adult worms were identified as *Echinostoma revolutum.*

Echinostomes (family *Echinostomatidae*) are intestinal trematodes of birds and mammals, including humans. Echinostomiasis can result in severe epigastric or abdominal pain accompanied by diarrhea, easy fatigue, and malnutrition (*1*). Heavy worm loads may lead to death due to intestinal perforation or marked malnutrition and anemia, as has been reported for infection caused by an echinostome species, *Artyfechinostomum malayanum* (under the name *Artyfechinostomum mehrai*), in India (*1*).

A total of 20 species of echinostomes that belong to 8 genera (*Echinostoma*, *Echinochasmus*, *Acanthoparyphium*, *Artyfechinostomum*, *Episthmium*, *Himasthla*, *Hypoderaeum*, and *Isthmiophora*) infect humans worldwide (*1*). *Echinostoma revolutum*, the most widely distributed species, is found from Asia and Oceania to Europe and the Americas (*1*). The first reported human infection was in Taiwan in 1929 (*2*). The prevalence of *E. revolutum* flukes in Taiwan during 1929–1979 varied from 0.11% to 0.65% (*3*). Small *E. revolutum*–endemic foci or a few cases of human infection were discovered in the People’s Republic of China, Indonesia, and Thailand until 1994 (*4**,**5*). However, no information is available about human *E. revolutum* infection after 1994, even in areas where the parasite was previously endemic.

In Cambodia, humans are commonly infected with intestinal nematodes and protozoa, including hookworms, *Strongyloides stercoralis*, *Ascaris lumbricoides*, *Trichuris trichiura*, and *Giardia lamblia* (*6**,**7*). However, with the exception of the blood fluke *Schistosoma mekongi*, infection with trematodes or cestodes has seldom been reported (*8*). Echinostomatid eggs have been detected in schoolchildren in 2 provinces, Battambang and Kampongcham (*9**,**10*), but adult worms were not collected for identification. The Korea Association of Health Promotion, South Korea, and The National Institute of Malaria, Entomology, and Parasitology, Ministry of Health, Cambodia, have been conducting an international collaboration to control intestinal helminthiases in schoolchildren in Cambodia (2006–2011). In June 2007, we conducted a fecal survey in 4 primary schools in Pursat Province, Cambodia, and found that an average of 11.9% of schoolchildren had positive test results for echinostome eggs. Adult worms recovered after the children received treatment with praziquantel and underwent purgation with magnesium salts were identified as *E. revolutum*. We report echinostomiasis as an endemic trematode infection among schoolchildren in Pursat.

## The Study

**Figure 1:**
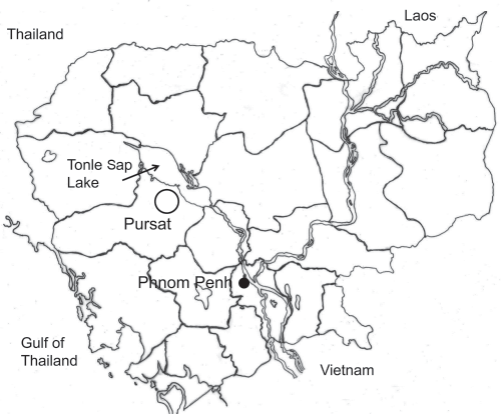
Surveyed area (circle) near Tonle Sap Lake, Pursat Province, Cambodia.

The surveyed areas were lakeside (the Tonle Sap Lake) villages in Pursat Province (Figure 1) where ≈12,000 persons, including 3,500 schoolchildren, live. For this study, 471 children (237 boys), 10–14 years of age, from 4 primary schools were selected. One fecal sample from each child was collected in June 2007. Samples were transported to the Malaria Station in Pursat within 2–3 days of collection and stored at 4°C until examination. The Kato-Katz thick smear technique was used to detect helminth eggs. Examination of feces and anthelmintic treatment were officially approved by the Ministry of Health, Cambodia, under the agreement of the Korea-Cambodia International Collaboration on Intestinal Parasite Control for Schoolchildren in Cambodia.

Four children who had positive test results for echinostomatid eggs and who had occasional, vague abdominal pain and discomfort were selected for anthelmintic treatment and adult worm recovery at the Malaria Station. After we obtained consent from their parents and the school guardian, the children’s infections were treated with a single oral dose of 10 mg/kg praziquantel (Shinpoong Pharmceutical Co., Seoul, South Korea), and purged with 20 g magnesium sulfate. Whole diarrheic feces were collected 3–4 times and pooled individually. The diarrheic feces were processed as previously described (*11*). Worms were collected by using a wooden applicator and washed several times in water. They were fixed with 10% formalin under coverslip pressure, stained with acetocarmine, and identified by morphologic features.

**Figure 2:**
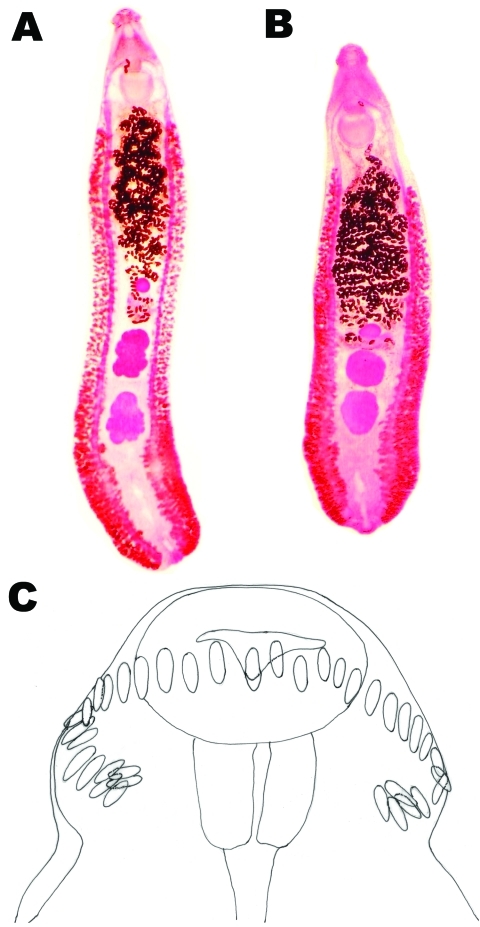
*Echinostoma revolutum* specimens recovered from schoolchildren in Pursat Province, Cambodia, which had 2 testes in the postequatorial region. A) An adult worm (8 mm long) showing lobulated testes. B) Another adult worm showing globular testes. C) Head collar of an adult specimen armed with 37 collar spines arranged in a single row, including 5 end-group spines on each side.

A total of 17.4% of samples were positive for helminth eggs. Echinostomatid eggs were found most frequently, followed by hookworm and *Trichuris trichiura* eggs (Table 1). The percentages of echinostome eggs were significantly higher in school A than in schools B, C, and D (Table 1). However, prevalence did not differ significantly (p<0.01) between boys and girls (data not shown). A total of 20 echinostome adults (12, 3, 3, and 2 worms) were recovered from 4 children who showed 48–120 eggs per gram of feces (Table 2). The worms were leaflike, elongated (Figure 2), and an average of 8.8 mm long (8.0–9.5 mm) and 1.7 mm wide (1.2–2.1 mm) (n = 10). When first passed in the feces, they were pinkish red and coiled in a “c” or “e” shape. The eggs in uteri were an average of 105 μm long (97–117 μm) and 63 μm wide (61–65 μm) (n = 10). On the basis of these characteristics, the worms were identified as *E. revolutum* (Froelich, 1802) Looss, 1899.

**Table 1:** Prevalence of intestinal helminths among schoolchildren, Pursat Province, Cambodia, June 2007*

School	No. children examined	No. (%) positive results for helminth eggs; 95% CI egg positive rate				
		Echinostomes†	Hookworms	*Trichuris trichuira* eggs	Others‡	Total§
A	116	**26 (22.4); 14.8–30.0**	4 (3.4); 0.1–6.7	1 (0.9); 0.0–2.6	1 (0.9); 0.0–2.6	31 (26.7); 18.6–34.8
B	117	**12 (10.3); 4.8–15.8**	12 (10.3); 4.8–15.8	0	3 (2.6); 0.0–5.5	26 (22.2); 14.7–29.7
C	118	**9 (7.6); 2.8–12.4**	0	0	1 (0.8); 0.0–2.4	10 (8.5); 3.5–13.5
D	120	**9 (7.5); 2.8–12.2**	7 (5.8)	0	0	15 (12.5); 6.6–17.2
Total	471	56 (11.9); 9.0–14.8	23 (4.9); 3.0–6.8	1 (0.2); 0.0–0.6	5 (1.1); 0.2–2.0	82 (17.4); 14.0–20.8

*Determined by examination of feces using the Kato-Katz technique. **Boldface** indicates significant differences between schools A and B (p = 0.01); A and C (p = 0.004), and A and D (p = 0.004), as analyzed by *z* test.CI, confidence interval. †Most of these are presumed to be eggs of *Echinostoma revolutum* worms. ‡Includes eggs of *Enterobius vermicularis* (schools A and B) and *Hymenolepis nana* (school C) worms. ¶Total no. of schoolchildren positive for >1 helminth species.

**Table 2:** Recovery of *Echinostoma revolutum* worms from schoolchildren, Pursat Province, Cambodia, June 2007*

Child no.	Age, y	No. echinostome eggs/g†	No*. E. revolutum* specimens recovered‡
1	13	48	12
2	13	120	3
3	10	120	3
4	13	96	2

*All children were female. Fecal samples were collected individually 2–3 hours after administration of MgSO_4_. †Eggs/g of feces; amount in a typical sample was assumed to be 41.7 mg. ‡All recovered worms were adult worms that contained eggs.

The major sources of *E. revolutum* infection in humans are freshwater clams (*Corbicula producta*) in Taiwan and snails (*Physa occidentalis* or *Lymnaea* sp.) in Thailand (*1**,**5*). According to school personnel, the children were fond of eating undercooked snails or clams of unidentified species sold on the road to their homes after school. They stated that the mollusks are caught near Tonle Sap Lake. Reasons for the higher prevalence in school A than schools B, C and D are unclear.

## Conclusions

Of the schoolchildren living near Tonle Sap Lake, Pursat Province, Cambodia, who participated in this study, 7.5%–22.4%, depending on school, were infected with *E. revolutum*. *E. revolutum* trematodes are endemic parasites in this area of Cambodia and a likely source of infection is freshwater snails or clams from the lake. The public health significance of echinostomiasis and educational and prevention efforts should be highlighted.

Echinostomiasis is not only an endemic infectious disease in Asian countries, including Cambodia, but also can be imported by overseas travelers from the United States or Europe. An outbreak of echinostomiasis was reported among US travelers returning from Kenya and Tanzania, although the source of infection was uncertain (*12*). This diagnosis should also be considered in patients with abdominal pain and diarrhea who have traveled to Southeast Asia and eaten snails or clams.

Despite the dangerous nature of echinostomes, the study of echinostomiasis has been neglected for many decades (*13**,**14*), possibly because physicians and laboratory personnel lack knowledge about this trematode parasite. In addition, no easy diagnostic technique is available to detect echinostome eggs, except for routine fecal examination. However, some microscopists seem to overlook or misinterpret the presence of echinostome eggs, particularly in Kato-Katz fecal smears. Even if echinostome eggs are detected, the specific diagnosis is not possible unless the adult worm is collected and identified. Thus, both the training of microscopists and emphasis on the clinical significance of echinostomiasis are urgently needed.
